# Effect of Acute Lung Injury (ALI) Induced by Lipopolysaccharide (LPS) on the Pulmonary Pharmacokinetics of an Antibody

**DOI:** 10.3390/antib14020033

**Published:** 2025-04-06

**Authors:** Shweta Jogi, Dhaval K. Shah

**Affiliations:** Department of Pharmaceuticals Sciences, School of Pharmacy and Pharmaceutical Sciences, The State University of New York at Buffalo, Buffalo, NY 14214-8033, USA; sjogi@buffalo.edu

**Keywords:** antibody, pulmonary, lung, pharmacokinetics, ALI, LPS, antibody biodistribution coefficient, pathophysiology, BAL

## Abstract

Objective: To investigate the effect of Lipopolysaccharide (LPS)-induced acute lung injury (ALI) on the pulmonary pharmacokinetics (PK) of a systemically administered antibody in mice. Method: The PK of a non-target-binding antibody was evaluated in healthy mice and mice with intratracheal instillation of 5 mg/kg LPS. The plasma, bronchoalveolar lavage (BAL), trachea, bronchi, and lung homogenate PK of the antibody were measured following intravenous administration of 5 mg/kg antibody dose. Noncompartmental analysis was performed to determine AUC values. Antibody concentrations in all biological matrices were quantified using qualified ELISA. The effect of ALI on BAL albumin and total protein concentrations was also determined. BAL protein concentrations were corrected for dilution using plasma urea concentrations. Results: Intratracheal instillation of LPS and the resultant ALI led to ~2–4-fold higher concentrations of albumin and proteins in the BAL. LPS-induced ALI also notably altered the pulmonary PK of the antibody. The effect of ALI on the antibody PK was time and tissue dependent. The trachea and bronchi showed ~1.7-fold and ~1.4-fold lower antibody exposure compared with the control group, but the BAL fluid exhibited ~4-fold increase in antibody exposure following LPS treatment. Most noticeable changes in antibody PK occurred 24 h after LPS administration, and the effect was temporary for the bronchi and trachea. However, the changes in lung homogenate and, more notably, in BAL persisted until the end of the experiment. Thus, our investigation suggests that due to the acute nature of ALI-induced pathophysiology and the changing severity of the disease, the dose and timing of antibody administration following ALI may need to be optimized based on the target site of action (e.g., bronchi, trachea, BAL, lung parenchyma, etc.) to maximize the therapeutic effect of the antibody. Conclusions: ALI may significantly affect pulmonary PK of systemically administered antibodies. Changes caused by ALI are time and tissue dependent, and hence, the timing and dose of antibody following ALI may need to be optimized to maximize the therapeutic effect of the antibody at the site of action.

## 1. Introduction

The prevalence of lung disorders has steadily increased, influenced by factors such as tobacco smoke, pollution, and genetics [1]. In the U.S., approximately 200,000 acute lung injury (ALI) cases occur annually, with a 30–40% mortality rate, depending on severity and conditions [2]. ALIs pose a significant economic burden due to high ICU costs, prolonged hospital stays, and the need for long-term rehabilitation. ALI is associated with high morbidity and mortality, particularly among critically ill patients.

ALI can result from a variety of direct and indirect effects on the lung, including pneumonia, sepsis, aspiration of gastric contents, and trauma. The pathophysiology of ALI involves complex inflammatory processes, with the activation of immune cells and the release of inflammatory mediators that contribute to endothelial and epithelial damage within the lungs [1,3,4]. Regardless of improvements in critical care management, there are limited therapeutic options specifically aimed at the underlying mechanisms of ALI. Current treatment for ALI is primarily supportive and includes mechanical ventilation along with lung-protective strategies such as fluid management, surfactant therapy, inhalation of vasodilators like nitric oxide, use of anti-inflammatory agents like glucocorticoids, and the treatment of underlying conditions like sepsis [5,6,7,8,9]. While these supportive measures can improve patient survival, they do not directly address the underlying inflammatory and oxidative mechanisms that drive ALI pathology. Thus, there is a need to develop better therapeutic options to treat pulmonary disorders.

Protein-based therapeutics, such as monoclonal antibodies, can be used to treat pulmonary disorders such as ALI effectively [10,11,12]. Although significant efforts are being made to use biologics to treat pulmonary disorders, there is a lack of understanding of the pulmonary PK of these proteins. Protein therapeutics are primarily administered through the intravenous (IV) route, with alternative routes such as oral, buccal, nasal, inhalation, and transdermal delivery also in use. It is typically assumed that the systemic exposure of antibodies is higher than pulmonary exposure in healthy individuals [13]. However, there is a possibility that this might change with any pathophysiological changes that accompany ALI. It has been reported that bronchoalveolar lavage (BAL) albumin concentration increased ~8-fold after LPS-induced ALI. This was observed because of the increase in vascular permeability and other molecular-level changes in the epithelial membrane of the lung [14,15,16]. Therefore, it is possible that an increase in vascular permeability after ALI can also increase the entry of antibodies administered systemically in the lung. As such, one can hypothesize that BAL concentrations of therapeutic antibodies that are present in the systemic circulation can also increase during ALI. However, such a hypothesis needs to be validated using dedicated in vivo experiments. The protein therapeutics PK in pulmonary tissues in healthy conditions after systemic administration is well reported; however, there has been limited literature available that explores the protein distribution in the lungs under diseased conditions like ALI.

This study focuses on addressing the disposition of a representative non-binding antibody (i.e., trastuzumab) in lungs after IV administration in an LPS-induced ALI model. Since trastuzumab is an anti-human HER2 antibody, it does not interact with murine HER2, and it acts as a ‘typical’ non-target-binding antibody in mice, which helps us minimize any interference from target binding on the antibody PK. LPS is commonly used to model ALI in mice because it effectively induces a strong inflammatory response like what is observed in human ALI. LPS, a Gram-negative bacteria component present in the outer membrane, plays a critical role in triggering the immune response by binding to TLR4 (Toll-like receptor 4) and releasing proinflammatory cytokines and chemokines. This results in alveolar capillary barrier dysfunction, fluid accumulation in the lungs, and irregular gas exchange [17,18]. In this study, we have performed in vivo PK studies in two groups of mice (i.e., one as a control group and another as an LPS-treated group) to understand the difference in the biodistribution of antibodies in pulmonary tissues during the pathophysiological conditions in the lungs.

## 2. Materials and Methods

### 2.1. Materials

The primary and secondary antibodies for ELISA were purchased from Bethyl Laboratory (Montgomery, TX, USA). BCA Assay kit, 384-well plates, para-nitrophenyl phosphate (PNPP), and tetramethylbenzidine (TMB) reagents were purchased from Thermo Scientific (Waltham, MA, USA). Urea assay kits were purchased from LS Bio (Newark, CA, USA). Mouse bio-intubation kit was procured from Braintree Scientific (Braintree, MA, USA). Lipopolysaccharide (LPS), i.e., *Escherichia coli* O111:B4, was purchased from Sigma Aldrich (St. Louis, MO, USA).

### 2.2. Protein Production, Purification, and Characterization

Trastuzumab was produced by cloning VH and VL genes into our in-house antibody expression vector IGK-FRT. Transfection of the constructed plasmid was performed into the CHO cells using Lipofectamine^TM^ 3000 Transfection Reagent (Thermo Fisher Scientific, Waltham, MA, USA, cat# L3000015). Further, the transfected cells were screened for 2 weeks with 1 mg/mL of hygromycin and then expanded into CD CHO media for 3 weeks, as detailed before [19,20]. Protein purification was performed using Protein G Chromatography. The antibody was buffer-exchanged with PBS pH 7.0 buffer and stored at 4 °C. SDS-PAGE gel electrophoresis was used to confirm the individual peak in the chromatogram.

### 2.3. Development of ALI Model

The group of mice designated for inducing ALI was challenged by intratracheal instillation with a 5 mg/kg dose of LPS from *E. coli* O111:B4 dissolved in sterile PBS (1X) to induce chronic pulmonary inflammation. Intratracheal instillation was performed using Braintree’s bio-intubation kit for mice. First, the animals were anesthetized via isoflurane inhalation and positioned on the intubation platform, suspended by their maxillary incisors at a 90° angle to the ground (Figure S1). Using blunt-tipped forceps, the tongue was gently extended to clear the airway while being held outside with the subordinate hand. Using the optical fiber guide (Figure S2), the catheter was subsequently placed within the trachea, aligning with the mid axis of the animal [21,22]. The catheter’s position inside the trachea was verified by observing condensation around it from gasping or by introducing 10 µL of PBS and checking for its pulsatile movement caused by respiration.

Trypan blue dye was used in the initial experiments to check for the effectiveness of the intratracheal instillation process. The distribution of trypan blue in lungs and gastrointestinal (GI) tract was examined to assess the instillation’s effectiveness. Several attempts were made to ensure precise dosing into the lung airway while reducing the dye’s entry into the throat and abdomen. Sedated animals were dosed intratracheally with varying volumes of dye (50 to 100 µL of a 100-fold diluted Trypan blue solution in PBS). After dosing, the animals were inspected to check if trypan blue was present in the lungs and stomach, as shown in Figure S3. Respiration and activity of the animals were observed throughout the procedure. When successful intubation was achieved, LPS was then administered into the trachea using a syringe connected to the catheter. The animals were returned to their respective cages and kept under a heating lamp for their recovery post-surgery. All the mice that underwent instillation were monitored to check for changes in weight, body temperature, hydration, and movement both before and after the instillation.

### 2.4. In Vivo PK Study

**Figure 1:**
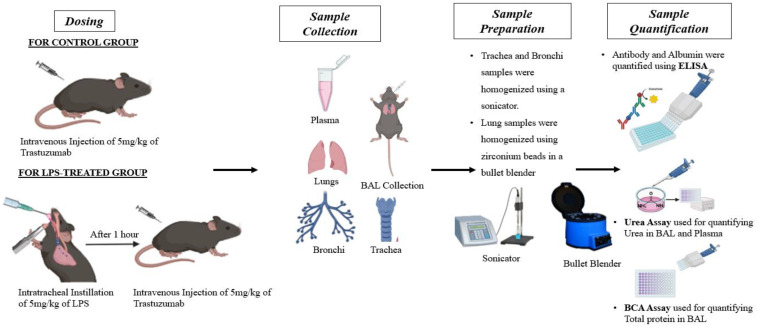
A graphical depiction of the experimental setup used to assess pharmacokinetics of trastuzumab in lung tissues for both the control and LPS-treated groups. C57BL/6J mice in the control group received a 5 mg/kg dose of trastuzumab. In the case of the LPS-treated group, C57BL/6J mice first underwent intratracheal instillation of LPS and then, after 1 h, were dosed with trastuzumab at 5 mg/kg dose. At specific time points, animals from both groups were euthanized, and samples, including plasma, lungs, BAL, bronchi, and trachea, were collected. The collected samples were processed and analyzed using ELISA. To account for dilution in the BAL fluid, a urea assay was conducted on both plasma and BAL samples. BCA assay was performed on BAL to quantify total protein concentrations in BAL fluid.

All in vivo PK studies were executed in wild-type male C57BL/6J mice in accordance with the IACUC protocol (PROTO202000054). All animals were housed individually under normal conditions with free access to food and water. The study design is illustrated in the accompanying figure (Figure 1). Two study groups were created, one as the control group and another as the LPS-treated group. Since preliminary investigation suggested no effect of intratracheal PBS instillation on antibody PK, the control group contained mice with no treatment. Investigation was carried out in a total of 60 mice, wherein 18 were dosed with the antibody, which represented the control group, and 25 belonged to the LPS-treated group. Three control group mice were euthanized at six distinct time intervals, resulting in a sample size of n = 3 for each interval. Animals were sacrificed at 5 min, 8 h, 12 h, 24 h, 72 h, and 7 days. Furthermore, 5 mice from the LPS-treated group were sacrificed at 5 different time points, giving n = 5 at each point to provide enough power to the study. Animals were sacrificed at 5 min, 1 h, 8 h, 24 h, 48 h, and 72 h after retro-orbital dosing. These timepoints for the LPS-treated group were determined based on the published literature [22,23,24], which indicates that LPS effects typically subside by 72 h.

The mice that belonged to the control group were sedated using isoflurane inhalation in an enclosed chamber and administered 5 mg/kg of antibodies systemically through retro-orbital injection. Plasma collection was performed via sub-mandibular sampling. The mice were then euthanized by cervical dislocation. BAL was collected after the animals were verified dead. The animal was positioned on its back on a surgical plate, with its limbs secured using pins. A scalpel was used to carefully cut the skin on the neck near the trachea. The muscle was cut around the trachea with pincers to expose it. Using pincers, a suture thread was carefully positioned beneath the trachea. A 26 G needle was carefully used to make a hole at the center of the exposed trachea, taking care to avoid any additional damage. Further, the catheter was inserted approximately 0.5 cm into the trachea, ensuring no damage to the lung. The catheter was secured by knotting the trachea around it with suture thread. A 1 mL syringe was attached to the catheter, which contained 1 mL D-PBS. The fluid was gently aspirated into the lung and slowly collected, giving us bronchoalveolar lavage fluid [25,26], which was then stored in a −80 °C freezer. The tissues were blotted to remove any remaining blood and then stored in a −80 °C freezer.

For the LPS-treated diseased group, mice were intratracheally instilled with a 5 mg/kg dose of Lipopolysaccharide (LPS) from *E. coli* O111:B4 dissolved in sterile PBS (1X). Intratracheal instillation was performed as mentioned earlier. One hour later, the animals received a 5 mg/kg dose of antibodies via retro-orbital injection. BAL and tissue collections followed the same procedure as in the control group.

### 2.5. Sample Preparation

Samples of all the pulmonary tissues were weighed and homogenized at a 1:4 *w*/*v* ratio using Pierce^®^ RIPA Buffer (Thermo Fisher Scientific) supplemented with 1 × Halt™ Protease Inhibitor Cocktail (Thermo Fisher Scientific). The lung tissue was homogenized with a BeadBug™ microtube homogenizer (Benchmark, Ames, IA, USA), utilizing seven 3.0 mm zirconium beads (Benchmark) in each tube. The homogenization process involved three cycles of 15 s each, with a 30 s cooling period on ice between cycles [27]. Due to the small volumes of trachea and bronchi tissues, these samples were homogenized with a Fisherbrand™ Model 120 Sonic Dismembrator (Fisher Scientific, Waltham, MA, USA). Each sample underwent three sonication cycles of 3 s on and 5 s off, with the amplitude set to 45% [28]. Once the tissues were homogenized, they were kept on ice for 2 h to equilibrate. Both tissue and BAL samples underwent centrifugation at 15,000× *g* for 15 min at 4 °C. The resulting supernatant was collected and analyzed using ELISA.

### 2.6. Analytical Method Development

#### 2.6.1. ELISA for Quantification of Antibody

The concentrations of antibody in tissues were quantified using ELISA. The quantification process followed a sandwich ELISA method consisting of several steps, as reported by Chang et al. [27]. Briefly, the steps involved the following: (a) a 384-well plate was coated with a primary antibody and left to incubate overnight, (b) nonspecific binding sites were blocked using blocking buffer, followed by incubation, (c) samples, standards, and quality controls (QCs) were added to the plate and incubated, (d) the secondary antibody was then added and incubated, and (e) then, the substrate was added and the plate was read. Standard curves were generated using 4-parameter logistic regression models. All samples were analyzed in triplicate, and the standard curves were generated in the relevant biological matrix (i.e., plasma, lung tissue, and BAL). Plasma samples were diluted 10,000 times, while tissue samples from the lungs, trachea, and bronchi were diluted 150 times in their respective matrices prior to analysis. BAL samples were diluted 5 times using a blank BAL matrix.

#### 2.6.2. Urea Assay

The antibody concentrations in the bronchoalveolar lavage samples were normalized using urea concentrations to account for sample dilution during BAL collection. Urea was measured in plasma and BAL samples using urea assay. All BAL data were normalized using plasma urea levels. A colorimetric urea assay kit (LS Bio LS-K331) was commercially sourced and utilized, following the steps as reported in Jagdale et al. [13]. The urea concentration in the samples was then calculated using the obtained data.

$$Urea\left(\frac{mg}{dL}\right)=\frac{ODsample-ODblank}{ODstandard-ODblank}*n*STD$$

Above, *OD*sample, *OD*blank, and *OD*standard are the optical density of the sample, standard, and blank at 520 nm wavelength, respectively. *STD* = 50 mg/dL for plasma and 5 mg/dL for BAL, and n = dilution for plasma and BAL samples. Samples with low urea levels, like BAL, were incubated for 50 min, whereas plasma samples with higher urea concentrations underwent a 20 min incubation. Dilution factor (DF) was calculated using the following equation.

$$Dilution\;factor=\frac{Plasma\;Urea\;Concentration}{BAL\;Urea\;Concentration}$$

#### 2.6.3. BCA Total Protein Assay

The total protein concentrations in BAL samples were analyzed using Thermo Scientific™ Pierce™ BCA Protein Assay Kit, which provides a detergent-compatible colorimetric method for detecting and quantifying total protein using bicinchoninic acid (BCA). The concentrations were determined using the following steps: (a) preparation of diluted albumin standards (20 to 2000 µg/mL) from Bovine Serum Albumin (BSA) stock of concentration 2000 µg/mL, (b) the BCA Working Reagent (WR) was prepared in a 50:1 ratio (reagent A:B), (c) each standard or unknown sample replicate (25 µL) was dispensed into a 96-well microplate (with a working range of 20–2000 µg/mL), (d) 200 µL of WR was added to each well and thoroughly mixed on a plate shaker for 30 s, (e) incubated at 37 °C for 30 min, (f) allowed the plate to cool to room temperature, (g) measured the absorbance at 562 nm and calculated the total protein concentration for unknown BAL samples.

#### 2.6.4. ELISA for Albumin Quantification

ELISA was used to measure albumin concentrations in BAL samples as per the steps outlined in Section 2.6.1. The capture antibody used was an anti-mouse albumin cross-absorbed and the detection antibody used was HRP-conjugated goat anti-mouse albumin antibody. Absorbance was measured at 650 nm. All samples were analyzed in triplicate, and the standard curves were generated in the relevant biological matrix (i.e., BAL).

### 2.7. Statistical Analysis

Results are presented as mean ± standard error of the mean (SEM). Differences between the LPS-treated group and control group were assessed for significance using one-way ANOVA, which was performed using GraphPad Prism (version 8.4.2). A value of *p* < 0.05 was considered statistically significant.

## 3. Results

### 3.1. Antibody Production and Purification

Trastuzumab, a non-target-binding antibody, was produced in-house, and the purity of the antibody was confirmed by looking at the sharpness of the peak in the absorbance vs. time plot generated during the purification process. A yield of approximately 15 mg/mL was obtained. SDS-PAGE was performed to confirm the purity of the antibody in reducing and non-reducing conditions, as shown in Figure S4. SDS-PAGE analysis showed > 95% purity with a significant single band corresponding to the intact antibody.

### 3.2. Development of Analytical Methods (i.e., ELISA, Urea Assay, and BCA Assay) for Quantification of Proteins

The standard curves for ELISA developed to determine antibody concentration in the control and LPS-treated groups are shown in Figure S5 and Figure S6, respectively. Curves for BAL, plasma, and lung matrix were developed separately. The lower limit of quantification (LOQ) for lung tissues and plasma samples was set at 100 ng/mL, and for BAL samples it ranged from 30 to 60 ng/mL based on the dilution factor.

Figure S7 shows urea standard curves for BAL and plasma. Absorbance at 520 nm showed a linear correlation with urea concentrations. All samples were analyzed in duplicate. The total protein concentrations in the BAL samples were measured using the BCA assay to evaluate changes in protein levels following LPS treatment. Figure S8 shows the BCA assay standard curve for BAL.

The albumin concentrations in BAL were measured using ELISA. The ELISA calibration curves for albumin quantification are shown in Figure S9. Standard curves utilizing the five-parameter equation had R^2^ values of ≥0.95 in the SoftMax^®^ Pro (v6)software.

### 3.3. Effect of LPS-Induced ALI on Albumin and Total Protein Levels in BAL

**Figure 2:**
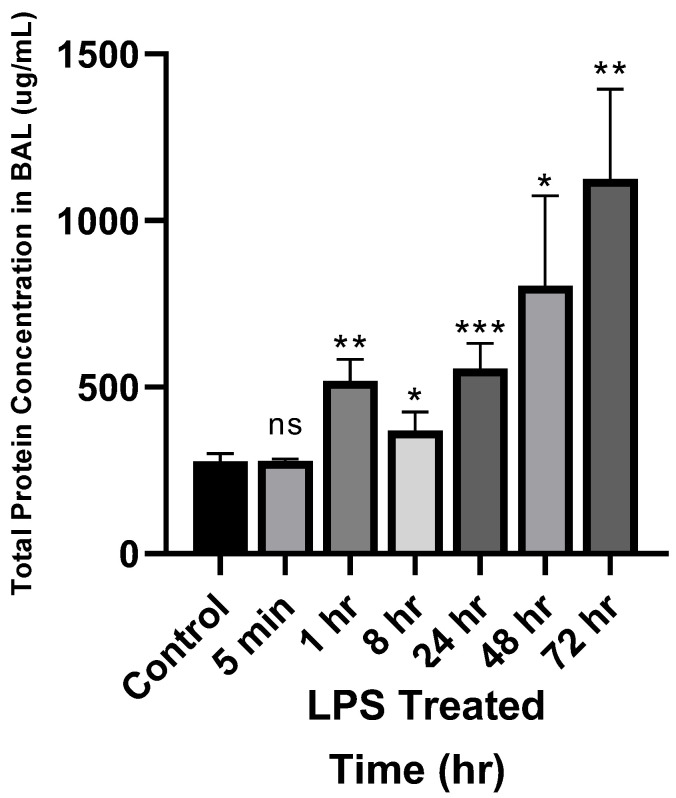
Effect of LPS treatment on total protein concentrations in BAL. Statistical significance performed using one-way ANOVA. ns = not significant; * *p* < 0.05; ** *p* < 0.01; *** *p* < 0.001.

Figure 2 shows the effect of LPS administration on the total protein levels in BAL. The results show that the LPS-treated group had higher protein concentrations compared with the control group. Specifically, the control group exhibited a protein concentration of approximately 300 µg/mL, whereas the LPS-treated group displayed a time-dependent increase in concentration ranging from 300 to 1400 µg/mL (i.e., ~4-fold higher). These findings confirm that LPS treatment promotes protein transfer into the epithelial lining fluid, and our ALI model is able to recapitulate the findings reported in the literature [29,30,31,32].

**Figure 3:**
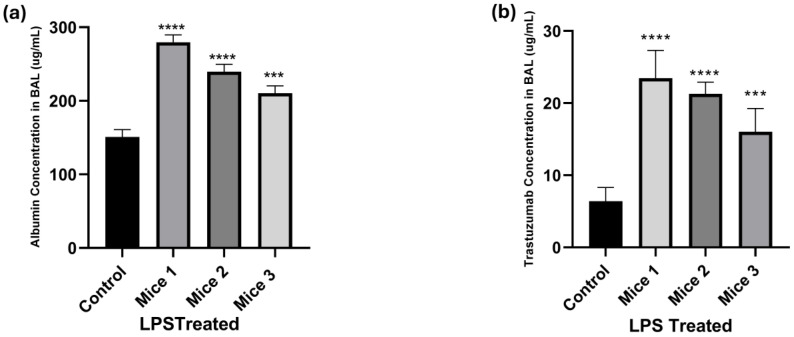
(**a**) Quantification of albumin in BAL 24 h after intratracheal instillation of LPS. (**b**) Quantification of trastuzumab in BAL 24 h after intratracheal instillation of LPS. Statistical significance performed using one-way ANOVA: *** *p* < 0.001; **** *p* < 0.0001.

Figure 3 shows the effect of LPS administration on albumin levels and antibody levels in BAL. It was observed that the control samples had an albumin concentration of 160 µg/mL, and in the LPS-treated group, it was in the range of 200–300 µg/mL (Figure 3a). The antibody concentrations observed for the control samples were 8 µg/mL, and in the LPS-treated group, they were in the range of 18–30 µg/mL (Figure 3b). Statistical analysis indicated significant differences in albumin and antibody concentrations between the control and the LPS-treated groups. These results show an increased concentration of albumin and antibody in the LPS-treated groups compared with the control group 24 h after LPS instillation. This observation confirms that LPS induces greater protein permeability across the epithelial lining fluid and shows that our ALI model is able to recapitulate the findings reported in the literature [29,31].

### 3.4. Effect of LPS-Induced ALI on Systemic and Pulmonary PK of Antibody

**Figure 4:**
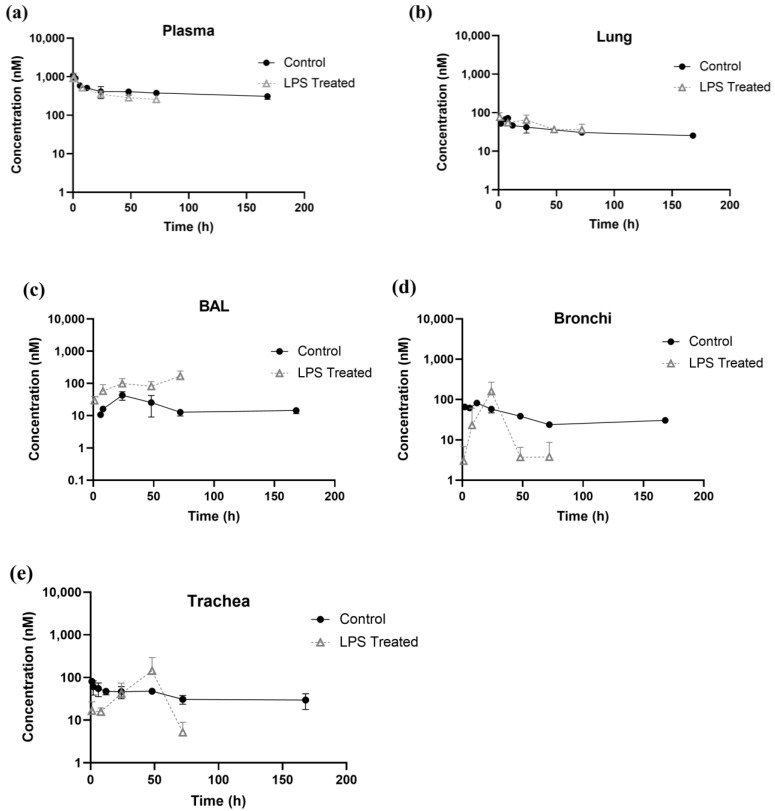
Pulmonary PK of antibody in control and LPS-treated groups after systemic administration of 5 mg/kg dose. Panels: (**a**) plasma, (**b**) lung homogenate, (**c**) BAL, (**d**) bronchi, and (**e**) trachea.

Figure 4 illustrates the pharmacokinetics of trastuzumab in the plasma, lung, BAL, bronchi, and trachea after a systemic administration of a 5 mg/kg antibody dose in both the control and ALI groups. The plasma exposure of the antibody was similar between the two groups (Figure 4a). In control group tissues, the peak concentration (C_max_) was observed at 6 h in the lung (Figure 4b), bronchi (Figure 4d), and trachea (Figure 4e), whereas in BAL (Figure 4c), the C_max_ occurred at 24 h, indicating a delayed distribution of antibody in BAL. In the control group, antibody exposure in the BAL was about 16 times lower than in the plasma, suggesting restricted penetration of the antibody from the systemic circulation into the epithelial lining fluid. The LPS-treated group exhibited delayed peak concentrations in the tissues, with C_max_ occurring at 24 h in the lung (Figure 4b) and bronchi (Figure 4d), at 48 h in the trachea (Figure 4e), and at 72 h in the BAL (Figure 4c). However, C_max_ for BAL may occur beyond 72 h, and further studies are required to confirm this observation. Notably, it was observed that the antibody exposure in BAL for the LPS-treated group was only ~3.5-fold lower than in the plasma (Figure 4c), which indicates that pathophysiological conditions such as the one observed in ALI could lead to higher exposure of systemically administered antibodies in the epithelial lining fluid of the lungs. As shown in Figure 4b, antibody exposure in the lungs of the LPS-treated group was slightly higher than in the control group as well. Conversely, antibody exposure in the bronchi and trachea was lower in the LPS-treated group compared with the control group, as shown in Figure 4d,e. Interestingly, the concentrations in the bronchi and trachea of the LPS-treated group at 48 h were higher than in the control group, suggesting the dynamic nature of pathophysiology induced by ALI and its effect on the pulmonary PK of the antibody.

**Figure 5:**
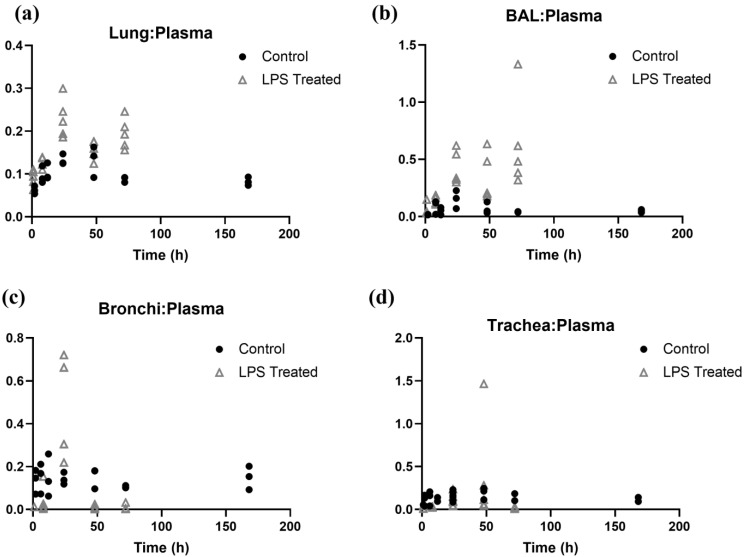
Tissue-to-plasma concentration ratio vs. time plot for control and LPS-treated groups. Panels: (**a**) lung homogenate, (**b**) BAL, (**c**) bronchi, (**d**) trachea.

In order to better understand how LPS-induced ALI influences tissue distribution of the antibody, tissue-to-plasma concentration ratios were plotted against the time and compared between the control and the LPS-treated group (Figure 5). A greater tissue-to-plasma concentration ratio was observed for lung and BAL in the LPS-treated group (Figure 5a,b). Conversely, in the case of bronchi and trachea (Figure 5c,d), it was observed that for most of the observation time points, the tissue-to-plasma concentration ratio was lower for the LPS-treated group. This observation suggests that the effect of ALI-induced pathophysiology is not monolithic across the lung, and different regions of the lung are affected differently when it comes to changes in the pathways responsible for the disposition of antibody in the lungs. At a few observation time points, antibody concentrations in the trachea (at 48 h) and BAL (at 72 h) exceeded those in plasma for a few animals. This may be due to significant pathophysiological changes in those animals or potential blood contamination during trachea or BAL sample collection procedures.

### 3.5. Effect of LPS-Induced ALI on Antibody Biodistribution Coefficient (ABC)

The antibody biodistribution coefficient represents the extent of tissue distribution for antibodies [33,34]. The biodistribution coefficient is calculated as the percentage ratio of tissue and plasma exposure of an antibody. The exposure and ABC value for trastuzumab after systemic dosing of antibody in the control and LPS-treated groups are shown in Table 1. The ABC value was approximately 14% for the lung, trachea, and bronchi in control tissues, aligning with previously reported values in the literature for healthy lung tissue [27]. Notably, a slightly higher distribution was observed in the trachea compared with the bronchi and lungs in the control group. In the LPS-treated group, the ABC values were approximately 16%, 10%, and 8% for the lung, bronchi, and trachea, respectively. It was observed that ABC values in the overall lung tissue were slightly elevated in the LPS-treated group, indicating increased antibody distribution within the lung tissue under inflammatory conditions. However, distribution within certain regions of the lung (i.e., bronchi and trachea) was reduced following LPS treatment. The trachea and bronchi showed approximately 1.7-fold and 1.4-fold lower ABC values, respectively, compared with the control group. Additionally, the BAL fluid exhibited ~4-fold increase in ABC values following LPS treatment.

## 4. Discussion

With the rising mortality associated with pulmonary disorders, compounded by the global impact of the COVID-19 pandemic, there is an increased focus on targeting lung tissues for treating various pulmonary diseases. Many of these diseases are driven by pathophysiological changes within the lung and may be addressed using protein-based therapeutics such as monoclonal antibodies. A thorough understanding of respiratory system anatomy and physiology is essential for investigating the etiology of these conditions and optimizing treatment strategies. The lungs exhibit distinctive anatomical and physiological structures that make them both promising for systemic drug delivery and challenging for localized drug administration. The respiratory system comprises the conducting zone (including the nasal cavity, trachea, bronchi, and bronchioles) and the respiratory zone (alveolar region). The lungs are characterized by a large epithelial surface area, a thin alveolar epithelium, and high vascularization. Epithelial cells within the lung are interconnected by tight junctions, forming a paracellular barrier that further restricts drug transport [35]. However, despite the good knowledge of lung anatomy and physiology, there is unawareness regarding determinants reliable for the disposition of protein therapeutics in various regions of the lung post systemic administration in a diseased condition. Thus, the development of protein therapeutics for pulmonary disorders remains empirical.

In this manuscript, we have presented an in vivo investigation that evaluates the impact of LPS-induced acute lung injury on the pulmonary disposition of an antibody. To facilitate a robust comparison of pulmonary PK under the pathophysiological conditions, an initial in vivo study was performed to establish the baseline pulmonary PK profile of the antibody in the healthy control group of mice. We observed that the ABC value for the lung homogenate was approximately 13.69%, which is similar to the reported value of ~15% in the literature [33]. ABC values in the bronchi and trachea were found to be similar to the lung homogenate. It was observed that BAL exposure of antibody was 3–4-fold lower than lung exposure, and the ABC value for BAL was only ~6%, suggesting minimal penetration of the antibody to the epithelial lining fluid from systemic circulation under healthy conditions. Jagdale et al. have also reported ABC values of antibody in mice lungs [13]. They found an ABC of 11.17% for lung tissue, and a slightly higher value of 15.22% for bronchi. They found an ABC of ~2% for BAL, and antibody exposure in BAL was found to be ~5–7-fold lower than the exposure in the lungs, trachea, and bronchi. Thus, their results were in line with our observations.

It is worth noting that BAL concentrations of antibody reported in our study are corrected for dilution. In addition to measuring antibody concentrations, urea levels were also quantified in both BAL and plasma samples to adjust for the dilution effects introduced during BAL collection. This dilution factor, determined via the urea assay, was applied to calculate corrected BAL antibody concentrations. Additionally, the bronchi tissue samples collected comprised early bronchioles rather than terminal bronchioles. In mice, bronchi tissues are extremely small (less than 5 mg), permitting only a single ELISA analysis per sample. Given the limited size of the trachea and bronchi in mice, obtaining enough blank tissue to generate a dedicated blank matrix for these tissues was challenging. As a result, the lung tissue matrix was used as a surrogate for protein quantification via ELISA in trachea and bronchi samples.

To model the ALI and associated pathophysiological conditions in mice, LPS was chosen as the endotoxin due to its capacity to replicate the condition effectively [36,37,38]. The concentration of LPS for intratracheal instillation was optimized to 5 mg/kg, based on the disease’s severity and symptoms as well as a review of various reported values [22,39]. LPS is a key proinflammatory glycolipid component found in the cell walls of Gram-negative bacteria, which are commonly present as a contaminant on airborne particles, such as those found in air pollution, organic dust, and cigarette smoke. Prolonged exposure to high LPS levels contributes to lung diseases like asthma, chronic bronchitis, and irreversible airflow obstruction, all marked by persistent inflammation [40,41]. Intratracheal instillation of LPS in mice induces several physiological changes, including histopathological alterations such as lung edema, alveolar hemorrhage, reduction in the expression of tight junction proteins, and thickening of the alveolar walls. Epithelial adhesion and barrier integrity are supported by intercellular structures such as tight and adherent junctions. ALI results in disruption of the paracellular permeability barrier in the alveoli. The barrier within the terminal airspaces is primarily maintained by tight junctions between alveolar epithelial cells, while vascular endothelial (VE) cadherin, an adhesion molecule at endothelial cell junctions, plays a crucial role in regulating vascular permeability and controlling leukocyte extravasation through VE cadherin-dependent adhesions [32]. As such, this process also triggers inflammatory cell infiltration, particularly involving polymorphonuclear granulocytes, and leads to the disruption of epithelial and endothelial cell structures. This damage in the endothelial and epithelial barriers in the lungs may also allow more proteins and fluids to leak into the alveolar spaces [32].

To confirm if our process of developing ALI by intratracheal instillation of LPS in mice is consistent with the literature reports, we first determined the effect of LPS administration on protein and albumin concentrations in BAL. Our results showed that following LPS administration, total protein concentration in BAL increased with time, and at 72 h, the protein concentration in BAL was about 4-fold higher than the control group (Figure 2). Similarly, albumin concentrations in BAL were also found to increase following LPS administration, and at 24 h, the albumin concentration in BAL was about 2-fold higher than the control group (Figure 3). Interestingly, the increase in total protein and albumin concentrations at 24 h after LPS administration was similar (i.e., ~2-fold). These results are reliable with reports in the literature that show that LPS causes an increase in BAL albumin concentrations [14]. Thus, it is safe to assume that the mouse model of ALI developed by us behaves similarly to the published models, and intratracheal instillation of LPS leads to increased levels of systemic proteins in BAL.

When investigating the PK of the antibody in LPS-treated mice, we observed that while plasma exposure of the antibody was similar to the non-treated mice, there was a tendency toward a slight reduction in the exposure (Figure 4a and Table 1). We hypothesize that this may be due to increased vascular permeability following LPS treatment, which could lead to increased extravasation and tissue distribution of antibody in the tissues. In accordance with this hypothesis, it was also observed that LPS treatment led to a slight increase in lung exposure of the antibody (Figure 4b and Table 1). However, the effect of LPS-induced pathophysiology was not the same on different regions of the lungs. It was found that LPS-treated mice had ~4-fold higher antibody concentrations in BAL (Figure 4c), which was similar to the effect of LPS observed on total proteins and albumin levels in BAL (Figure 2 and Figure 3). This observation suggests that the pathways responsible for antibody and protein disposition in BAL had most severe changes following LPS administration, and these changes could be related to increased permeability of the endothelial and epithelial barriers and/or altered fluid flow and immune repertoire of the alveolar region. For example, Vernooy et al. have reported that LPS exposure triggers the production of tumor necrosis factor (TNF)-α, interleukin (IL)-1β, and interferon-γ, which serve as markers for inflammatory changes in the tissues [24,42]. Thus, it is important to consider LPS exposure-related changes in immune cells as a contributing factor to changes in pulmonary PK of the antibody. Further studies are needed to distinguish the impact of pathophysiological changes from immune cell-mediated changes in the pulmonary PK of the antibody. Additionally, residual blood content in the lungs may also change due to LPS-induced ALI, resulting in the observed changes, which needs to be further investigated. Contrary to changes in the BAL and lung homogenates, it was found that exposure to the antibody in the bronchi and trachea was reduced following LPS treatment. While one may need to perform more mechanistic and regional PK studies to investigate this discrepant observation, we hypothesize that this may be due to enhanced mucociliary clearance in these tubular regions along with their different anatomical structures [43,44]. The lower antibody exposures in the bronchi and trachea also imply that ALI may hinder antibodies’ ability to reach effective concentrations in these regions, limiting their efficacy in treating infections or inflammatory conditions within the bronchi and trachea.

It is important to note that the changes in pulmonary PK of the antibody observed in the mouse model of ALI are dynamic. Since the pathophysiology caused by intratracheal instillation of LPS is time dependent, the changes in pulmonary disposition of the antibody are also time dependent. To objectively evaluate these changes, tissue-to-plasma concentration ratios were calculated at different time points (Figure 5). It was observed that the most noticeable changes in antibody distribution occurred 24 h after LPS administration, and the effect was temporary for the bronchi and trachea. However, the changes in lung homogenate and, more notably, in BAL, persisted until the end of the experiment (i.e., 72 h). These results show that the timing of antibody administration following ALI matters due to the acute nature of the pathophysiology and the changing severity of the disease. As such, depending on the target site of action (e.g., bronchi, trachea, BAL, lung parenchyma, etc.) the dose and timing of antibody administration following ALI may need to be optimized to maximize the therapeutic effect of the antibody.

## 5. Conclusions

This study demonstrates that ALI may affect the pulmonary PK of systemically administered antibodies. Significant increases in BAL antibody concentrations were observed, which could be advantageous following lung injury if the site of action is in the epithelial lining fluids. On the contrary, antibody exposure in the bronchi and trachea was reduced, which may be detrimental if the site of action is in the tubular region of the lungs. It was observed that the changes caused by ALI are time and tissue dependent, and hence, the timing and dose of the antibody following ALI may need to be optimized to maximize the therapeutic effect of the antibody at the site of action. The findings from this investigation can be expanded to other protein-based therapeutics (i.e., antibody derivatives) and should be verified in other animal species before being translated to the clinic. The novel PK data generated here also provide a foundation for the development of a physiologically based PK (PBPK) model for diseased lungs [45,46,47].

## Figures and Tables

**Table 1:** AUC_0-*last*_ (nmol*h/L) and ABC values for trastuzumab in lungs, BAL, bronchi, and trachea in control and LPS-treated groups.

	Control		LPS Treated	
Tissue	AUC_0-*last*_	ABC	AUC_0-*last*_	ABC
Plasma	5.03 × 10^4^		4.42 × 10^4^	
Lungs	6.89 × 10^3^	13.69	8.62 × 10^3^	15.88
BAL	3.09 × 10^3^	6.16	1.28 × 10^4^	23.67
Bronchi	6.92 × 10^3^	13.76	5.24 × 10^3^	9.65
Trachea	7.04 × 10^3^	14.02	4.39 × 10^3^	8.09

## Data Availability

The datasets generated in the current study are available from the corresponding author upon reasonable request.

## Supplementary Materials

The following supporting information can be downloaded at: https://www.mdpi.com/article/10.3390/antib14020033/s1, Figure S1: Intra-tracheal Instillation experimental setup to induce Acute Lung Injury. Figure S2: The mice were intubated using a catheter guided by an optical fiber to ensure accurate intra-tracheal instillation. Figure S3: Lung tissue following intratracheal instillation of trypan blue, with the presence of blue dye confirming successful pulmonary delivery. Figure S4: SDS-PAGE: In reducing condition, bands observed at 25kDa and 50kDa and the absence of other bands confirm the purity of the antibody. In non-reducing conditions, a dark band can be seen around 150kDa). Figure S5: ELISA Standard Curves for Control Group A) BAL Standard Curve for Trastuzumab B) Plasma Standard Curve for Trastuzumab C) Tissue Standard Curve for Trastuzumab. Figure S6: ELISA Standard Curves for LPS-Treated Group A) BAL Standard Curve for Trastuzumab B) Plasma Standard Curve for Trastuzumab C) Tissue Standard Curve for Trastuzumab. Figure S7: Urea Assay Standard Curves for BAL and Plasma. Figure S8: BCA Assay Standard Curves for BAL. Figure S9: Albumin ELISA Standard Curve for BAL.
