# Supplementary material for: Effect of Acute Lung Injury (ALI) Induced by Lipopolysaccharide (LPS) on the Pulmonary Pharmacokinetics of an Antibody
Source: Antibodies (Basel). 2025 Apr 6;14(2):33. doi: 10.3390/antib14020033 (PMC12015819; doi:10.3390/antib14020033)
Supplement: Supplementary file 1 [file antibodies-14-00033-s001.zip › antibodies-3516299-supplementary.pdf]

Supplementary Material

Effect of Acute Lung Injury (ALI) Induced by Lipopolysaccharide (LPS) on the Pulmonary Pharmacokinetics of Antibody

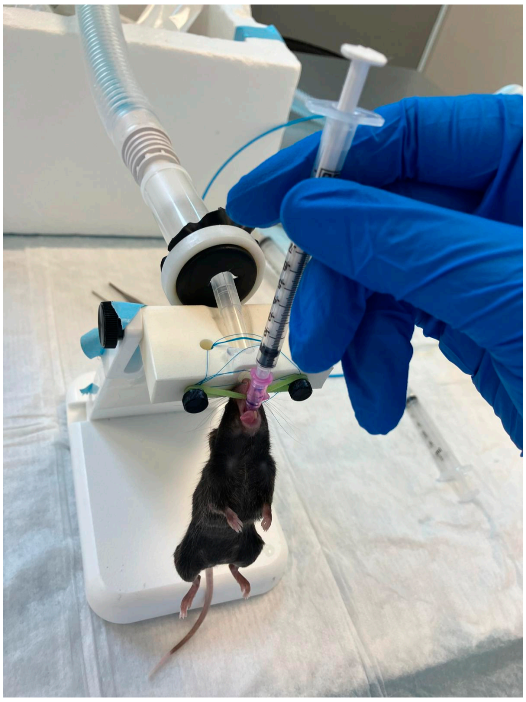

Figure S1: Intra-tracheal instillation experimental setup to induce Acute Lung Injury.

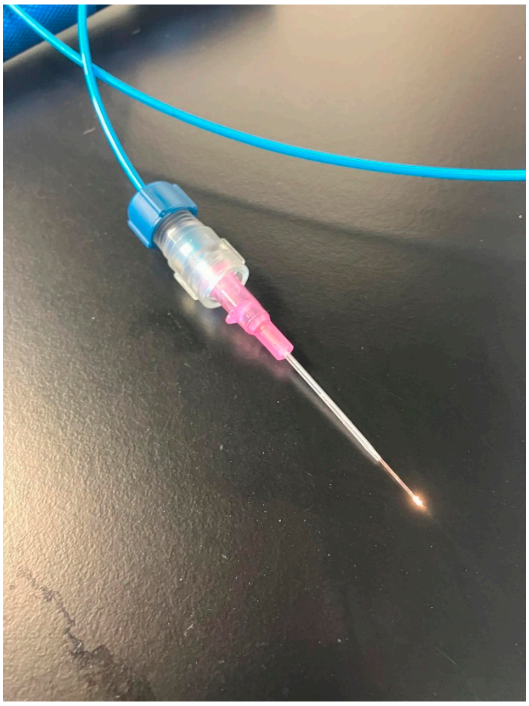

Figure S2: The mice were intubated using a catheter guided by an optical fiber to ensure accurate intra-tracheal instillation.

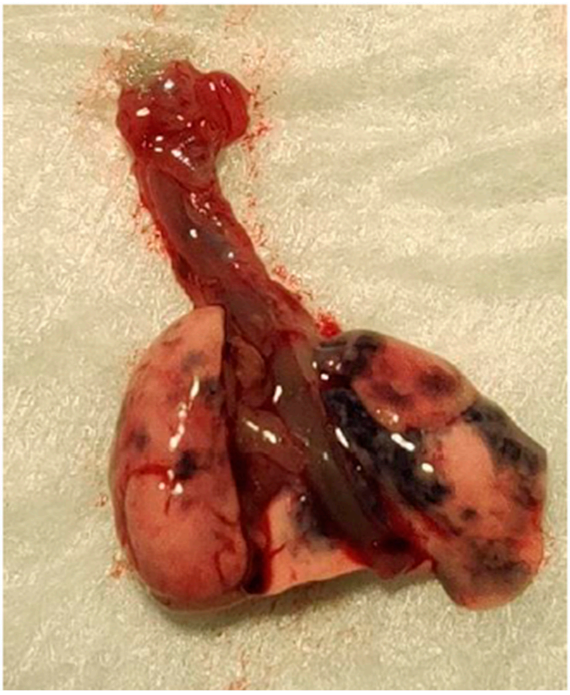

Figure S3: Lung tissue following intratracheal instillation of trypan blue, with the presence of blue dye confirming successful pulmonary delivery.

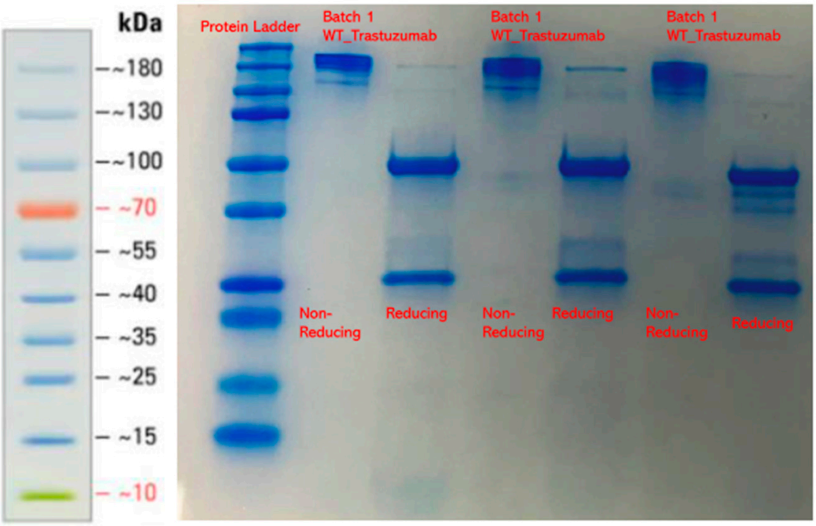

Figure S4: SDS-PAGE analysis. In reducing conditions, bands are observed at 25kDa and 50kDa and the absence of other bands confirms the purity of the antibody. In non-reducing conditions, a dark band can be seen around 150kDa.

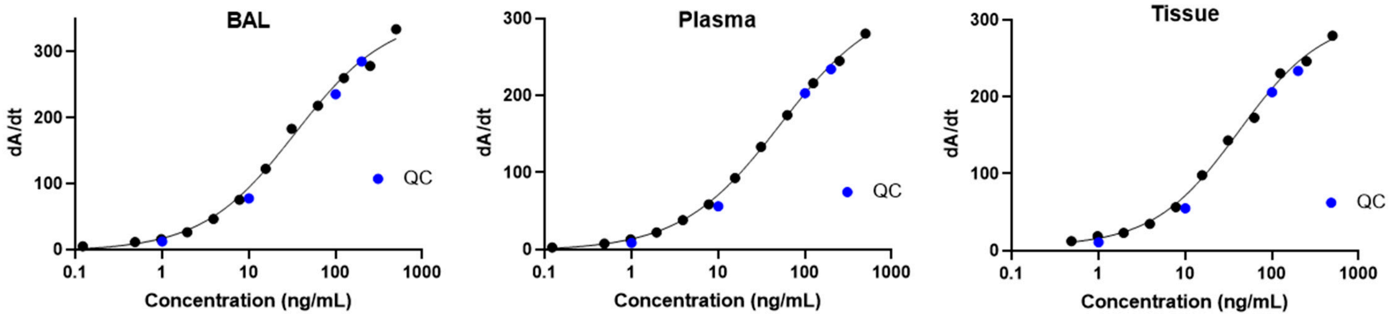

Figure S5: Standard curves for the quantification of antibody in BAL, plasma, and lung tissue samples of control group mice.

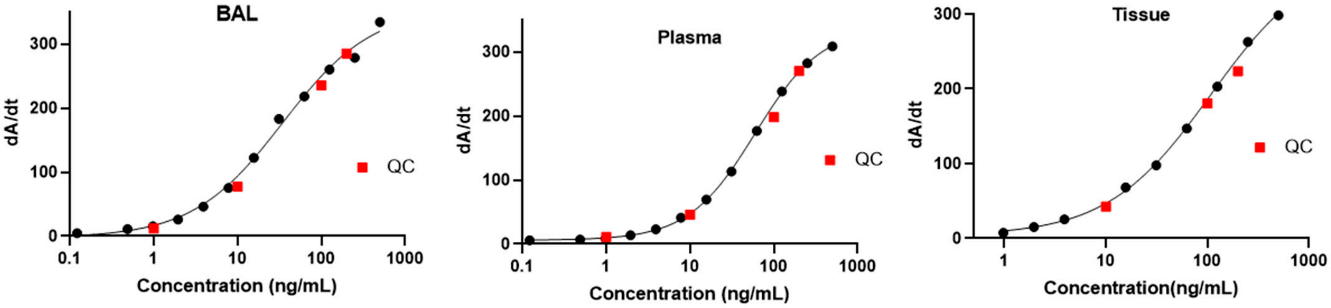

Figure S6: Standard curves for the quantification of antibody in BAL, plasma, and lung tissue samples of LPS-treated mice.

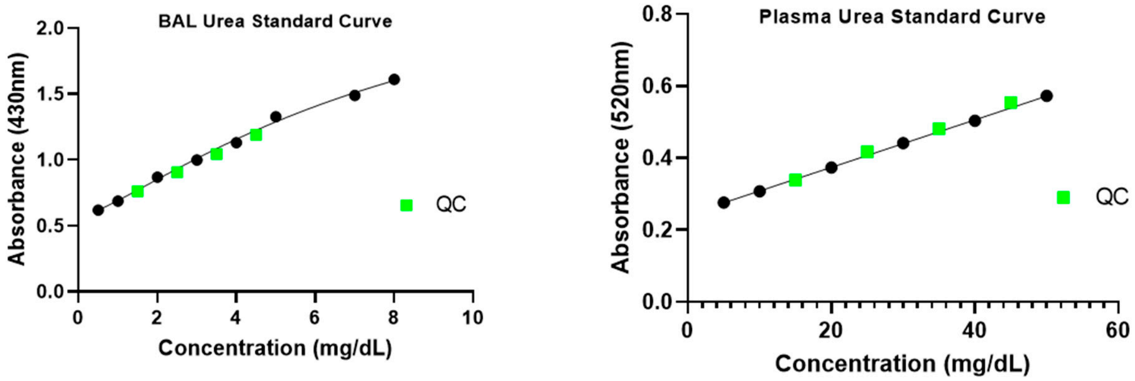

Figure S7: Standard curve for urea assay in BAL and plasma.

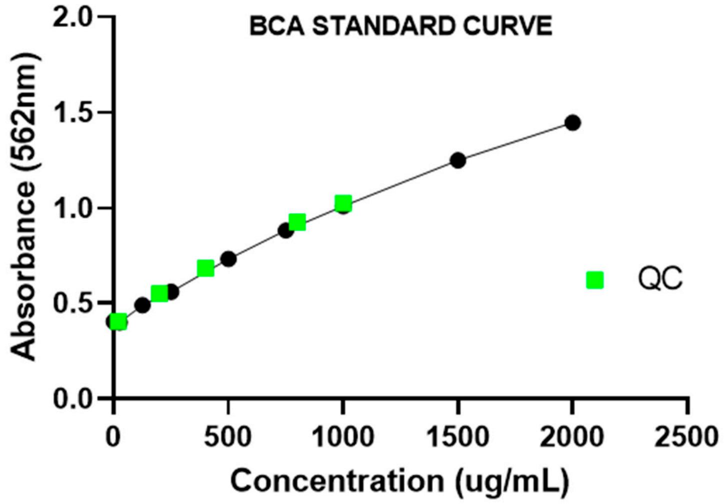

Figure S8: Standard curve for the BCA assay to quantify total protein concentration in BAL.

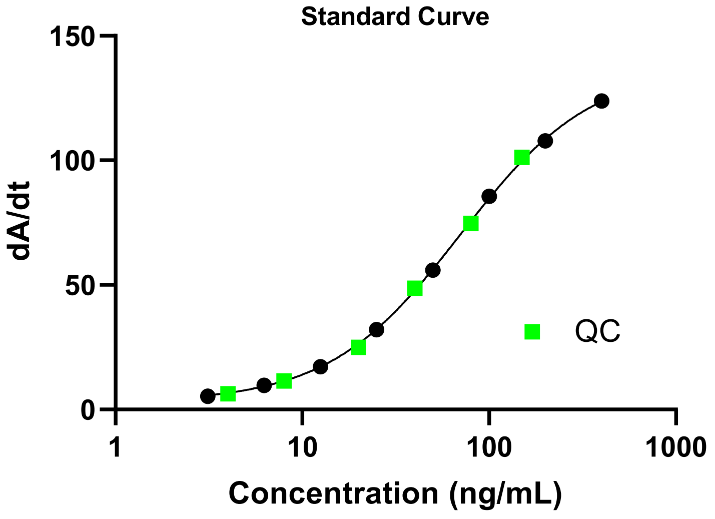

Figure S9: Standard curve for the ELISA to measure albumin concentrations in BAL.
